# Therapeutic Potential of Extracellular Vesicles for the Treatment of Nerve Disorders

**DOI:** 10.3389/fnins.2019.00163

**Published:** 2019-03-05

**Authors:** Luisa R. Galieva, Victoria James, Yana O. Mukhamedshina, Albert A. Rizvanov

**Affiliations:** ^1^Institute of Fundamental Medicine and Biology, Kazan Federal University, Kazan, Russia; ^2^School of Veterinary Medicine and Science, University of Nottingham, Nottingham, United Kingdom; ^3^Department of Histology, Cytology, and Embryology, Kazan State Medical University, Kazan, Russia

**Keywords:** extracellular vesicles, immunomodulatory effects, neuroprotective effects, mesenchymal stem cells, nerve disorders

## Abstract

The use of extracellular vesicles (EVs) as cell free therapy is a promising approach to stimulate tissue regeneration including that of the nervous system. EVs transfer bioactive proteins and lipids, RNA and microRNAs, which play a relevant role in EV-mediated intercellular communication. The immunomodulatory, anti-inflammatory, and neuroprotective effects of mesenchymal stem cells-derived EVs have been well studied, knowledge of this paracrine mechanism and the availability of these cells, positions mesenchymal stem cells as a potential source of EVs for cell free therapy for a variety of regenerative and nervous system disorders. In this review, we focus on the immunomodulatory and neuroprotective effects of stem cells-derived EVs within *in vitro* and *in vivo* models of nerve disorders.

## Introduction

The need to improve the effectiveness of available therapeutic protocols used within the regenerative medicine field, has led to a number of different approaches. However, the nerve disorders still present a treatment challenge due to the low regenerative potential of the central nervous system (CNS). Stem cell therapy has been at the forefront of regenerative medicine for the last decade, but poor clinical trial reports have shifted the focus of researchers to develop improved protocols and complementary approaches. For example, it became apparent, that the therapeutic effect of stem cells can be enhanced through the expression of genes of neurotrophic factors using gene therapy approaches. Unfortunately, a hazard associated with inducing such genetic changes is the potential for stem cell transformation. Therefore, a critical need still exists for the development of new therapeutic approaches that do not carry the same potential risks.

Confirmation of the paracrine effects of stem cells and the relevance to tissue regeneration was a breakthrough in this area, paving the way for a series of subsequent studies to characterize and leverage this mechanism for therapeutic development. Gnecchi et al. were the first to show that conditioned medium from mesenchymal stem cells (MSCs) overexpressing the gene *Akt1* exerted cytoprotective effects on cardiomyocytes exposed to hypoxia *in vitro*, reduced acute myocardial infarction sizes and improved ventricular functions *in vivo* (Gnecchi et al., 2005, 2006). Takahashi et al. (2006) similarly found that injection of bone marrow mononuclear cells supernatants increased microvessel density and decreased the fibrotic area within an infarcted heart model, contributing to significant functional improvement. Subsequently, MSCs-derived conditional medium was reported to stimulate functional and morphological improvement in pathological conditions such as renal injury, myocardial infarction, and fulminant hepatic failure (Bi et al., 2007; Parekkadan et al., 2007; Timmers et al., 2007).

The results obtained supported a paracrine hypothesis of stem cell action in tissue protection and repair. It was subsequently established that these beneficial were attributable to soluble factors including extracellular vesicles (EVs) released from stem cells (Bruno et al., 2009; Lai et al., 2010; Xin et al., 2013a). The biogenesis and methodology of isolation of EVs has been described in several published reviews (Biancone et al., 2012; Akers et al., 2013; Koniusz et al., 2016). Therefore, in this mini review, we focus on the newly emerging field investigating the therapeutic potential of stem cell-derived EVs, produced *in vitro* and transplanted within the animals and patient for the treatment of nerve disorders.

## Immunomodulatory and Neuroprotective Effects of Stem Cell-Derived EVs

By transferring bioactive proteins and lipids, RNA and microRNAs (miRNAs), EVs may play a crucial role in intercellular communication between stem cells and other cells within the tissue micro-environment (Camussi et al., 2013; Braccioli et al., 2014; Gomzikova and Rizvanov, 2017). The anti-inflammatory and neuroprotective effects of MSC-derived EVs and their paracrine mechanism of action have been well studied (Kalani et al., 2014; Börger et al., 2017).

EVs have been shown to be actively involved in processes of immune regulation such as stimulation of T-cell proliferation, apoptosis induction in activated cytotoxic T-cells, differentiation of monocytes into dendritic cells and B lymphocyte-mediated tumor suppression (Bobrie et al., 2011; Chaput and Théry, 2011; Taylor and Gercel-Taylor, 2011). It is not surprising that MSCs-derived EVs were also found to be immunologically active (Blazquez et al., 2014; Zhang et al., 2014; Bruno et al., 2015). The immunomodulatory properties of MSCs-derived EVs are reported to be the result of the suppression of TNF-a and IL-1b secretion, increasing the concentrations of TGF-b, regulatory T cells (Treg) and cytotoxic T lymphocyte-associated protein 4, inducing Th1 to Th2 cell conversion, reducing IFN-γ production and the potential of T cells to differentiate into Th17 cells (Mokarizadeh et al., 2012; Blazquez et al., 2014; Del Fattore et al., 2015; Chen et al., 2016b). However, some data indicate a lower *in vitro* immunomodulatory effect of MSC-derived EVs on T-cell proliferation and antibody formation, as compared with their cellular counterpart (Conforti et al., 2014). In a recent study, immunomodulatory effect inflammation-stimulated (TNF-α and IFN-γ) MSCs-derived EVs was evaluated (Harting et al., 2018). It was found, that thus obtained EVs have enhanced anti-inflammatory properties partially due to COX2/PGE2 pathway alteration.

Although EVs can exhibit a variety of different protein and nucleic acid cargos, their regenerative effects are mainly ascribed to the transfer of specific proteins and miRNAs (Collino et al., 2017). For example, EV cargos of miR-1000, miR-133b, miR-21, miR-34a, and miR-219 have been reported to exert a neuroprotective effect by regulating glutamate release at the synapse (Verma et al., 2015), promoting neural plasticity (Xin et al., 2013b), suppressing apoptosis (Ma et al., 2013; Vallabhaneni et al., 2015), and enhancing myelination (Pusic and Kraig, 2014b).

Despite knowledge of the function of some aspects of the EV cargo, further studies are required to fully understand the mechanisms that underpin the immunomodulatory and neuroprotective effects of EVs.

## Current Perspectives of EVs in the Treatment of Nerve Disorders

Although the therapeutic potential of EVs has not been fully elucidated, their use can be said to be most promising for the treatment of different disorders of the CNS, when drugs are of limited use due to their inability to penetrate the blood-brain barrier (BBB). EVs have been proposed to be transported across the interior of a cell via transcytosis, which may enable these EVs to cross the BBB. This has been experimentally confirmed, as EVs given intravenously (Alvarez-Erviti et al., 2011) or intranasally (Zhuang et al., 2011; Haney et al., 2015) were able to penetrate the BBB.

It should be noted that the therapeutic potential of EVs depends on the composition of their cargo. The cargo of EVs from different cell types can include a common set of biological molecules (nucleic acids, proteins, and lipids) and also molecules that reflect the cell source of the EVs and the physiological or pathological state of the cell source (Zhang et al., 2016; Reiner et al., 2017). In this regard, it is important to define the cargo of stem cell-derived EVs to further understand the mechanisms involved in regenerative processes during therapy. Currently, most studies evaluating the regenerative potential of stem cell-derived EVs focus on a general assessment of therapeutic efficacy without detailing the multiple physiological or biochemical changes occurring due to EVs specific cargo. Defining the EV cargo would allow subsequent manipulation, combining a specific set of proteins and RNAs which could efficiently modulate the course of nerve disorders (Selmaj et al., 2017).

**Figure 1:**
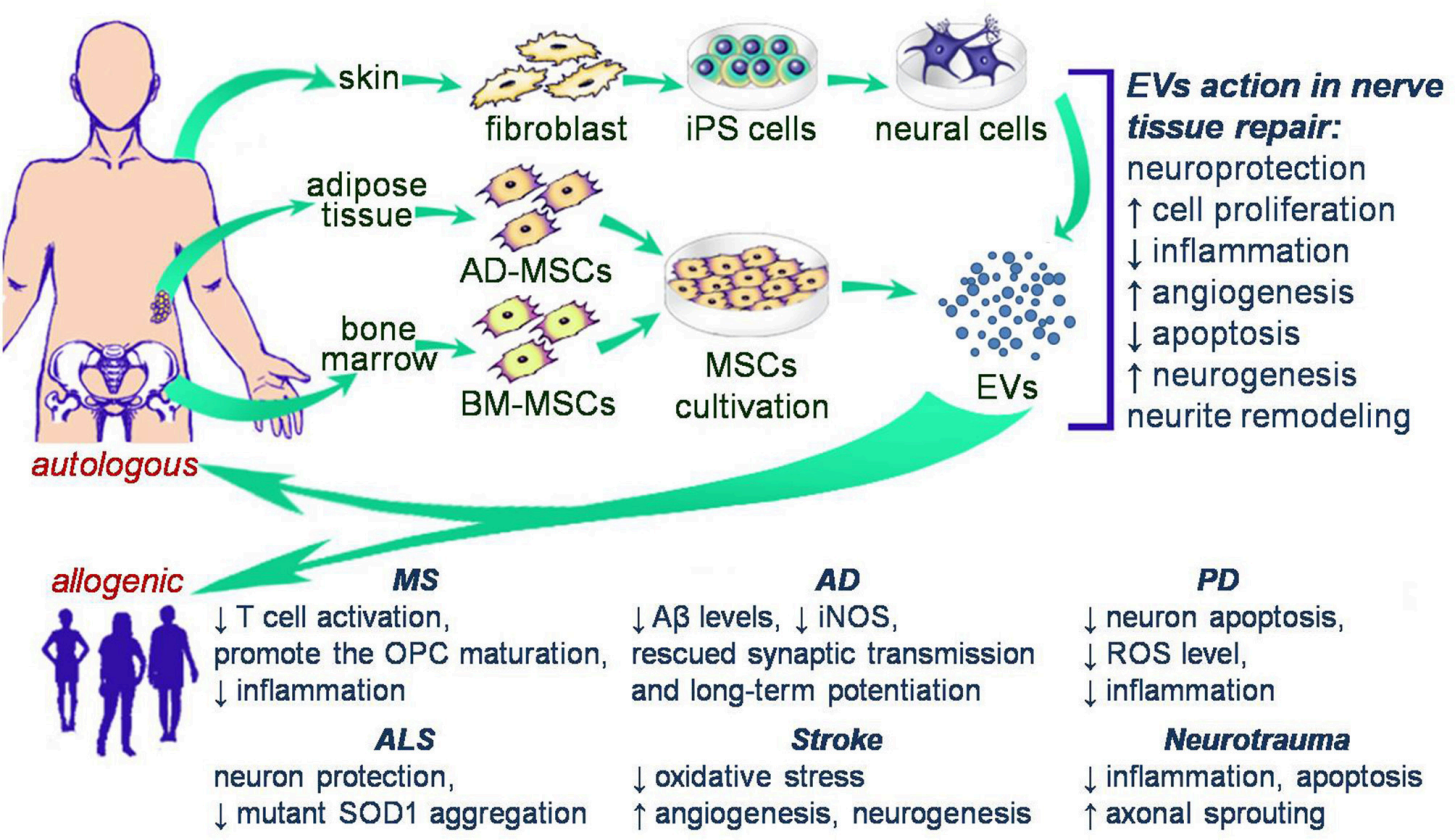
Schematic illustration of potential cell free therapy in clinical trials of nerve disorders.

The introduction of EV use into clinical practice has been limited by the ability to isolate sufficient EVs from culture systems and the heterogeneity of the cargo of naturally occurring EVs. Therefore, methods of obtaining “modified” EV have become a major focus for the field. In such a cases, EVs are most often derived from MSCs owing to the availability of these cells and the presence of positive properties mediated by their EVs, or induced pluripotent stem cells (iPS cells) whose EVs also demonstrate consistent regenerative abilities. EVs from both cell types have been proposed as a potential starting point for cell free therapy in nerve disorders (Figure 1).

Table 1 provides a summary of preclinical studies of EV use in both *in vitro* and *in vivo* models of nerve disorders.

**Table 1:** Preclinical trials using EVs.

**Disease model**	**Source of EVs**	**Protocol details**	**Reported effects**	**Reference**
**MULTIPLE SCLEROSIS**				
EAE in mice	EVs from bone marrow-derived DCs infected Ad/mTGF- 1 or Ad/sTGF-β1, ~100 nm	10 μg EVs/mouse, intravenously	Prevented the *de novo* differentiation of Th17 cells via inhibiting IL-6 production, inhibited the development and progression of EAE	Yu et al., 2013
Primary OPC cultures	serum-derived EVs of pregnant and non-pregnant mice	Co-culture of primary OPC with EVs, for 72 h	Enhanced the function of OPCs	Williams et al., 2013
EAE in mice	~100 nm	40 μg total protein of EVs/mouse, intravenously	Suppressed T cell activation, promoted the maturation of OPC, facilitated OPC migration into active CNS lesions	
Brain tumor-bearing mice model	Tumor cells-derived EVs with circumin or JSI124, ~100 nm	12.5 pmol EVs, intranasally for 12 consecutive days	Significantly delayed brain tumor growth in the brain tumor model; protected against LPS-induced brain inflammation and the progression of EAE	Zhuang et al., 2011
LPS-induced brain inflammation		1.5 nmol EVs in 10 μl PBS/mouse, single intranasal administration		
EAE in mice		1.5 nmol EVs, intranasally for 26 consecutive days		
**ALZHEIMER'S DISEASE**				
N2a cells	AD-MSC-derived EVs, ~100–200 nm	Co-culture of N2a cells with EVs, 500 μg protein/mL, up to 24 h	Decreased extracellular and intracellular Aβ levels in N2a cells	Katsuda et al., 2013
APP transgenic mice	Primary neuron culture-derived EVs	2 mg total protein of EVs/ml PBS by Alzet minipump at 0.25 ll/h for 14 days.	decreased Aβ and amyloid depositions	Yuyama et al., 2015
Primary cortical neuron culture from newborn APP/PS1 mice	BM-MSC-derived EVs, ~100–140 nm	Co-culture of cortical neurons with EVs, 100 μg/ml EVs for 12 h	Reduced Aβ induced iNOS expression in primary neurons	Wang et al., 2017
APP/PS1 mice		100 μg EVs in 5 μl PBS/mouse, i.c.v. injection once per 2 days for 2 weeks	Improved cognitive behavior, rescued impairment of CA1 synaptic transmission, and long-term potentiation	
**PARKINSON'S DISEASE**				
6-OHDA induced apoptosis in DA neurons	SHEDs-derived EVs, ~50–1000 nm	Co-culture of DA neurons with EVs, for 20 h	Suppressed 6-OHDA induced apoptosis	Jarmalavičiute et al., 2015
Cortical neurons and DA neurons from mouse pups	Macrophages—derived EVs incorporating therapeutic protein catalase, ~100–200 nm	Co-culture of cortical neurons and DA neurons with EVs, 100 μg/mL total protein, for 24 h	Reduced ROS level	Haney et al., 2015
6-OHDA lesion of C57BL/6 mice		2.4 × 10^10^ EVs in 20 μl PBS/mouse, intranasally or intravenously	Anti-inflammatory effect, reduced microgliosis	
**AMYOTROPHIC LATERAL SCLEROSIS**				
NSC-34 cells expressing ALS mutations	ADSC-derived EVs, 30–120 nm	Co-culture of NSC-34 cells with 200 μg/ml EVs for up to 18 h	Protected cells from oxidative damage	Bonafede et al., 2016
G93A primary neuronal cell culture	AD-MSC-derived EVs, ~100 nm	Co-culture of G93A cells with 200 μg/ml EVs, twice on day 2 and day 6	Reduced mutant SOD1 aggregation	Lee et al., 2016
**STROKE**				
MCAO in rats	BM-MSC-derived EVs, ~100 nm	100 μg total protein of EVs in 500 μl PBS/rats, intravenously	Improved functional recovery and enhanced neurite remodeling, neurogenesis, and angiogenesis	Xin et al., 2013a
MCAO in mice	BM-MSC-derived EVs, ~100 nm	EVs released by 2 × 10^6^ MSCs diluted in 250 μl of saline, intravenously	Reduced post-ischemic motor coordination impairment, induced long-term neuroprotection, increased cell proliferation, stimulated neurogenesis, and angiogenesis	Doeppner et al., 2015
MCAO in rats	AD-MSC-derived EVs, ~100 nm	100 μg EVs/rat, intravenously	Reduced infiltration of CD11^+^ and CD68^+^ cells, decreased oxidative stress, increased angiogenesis	Chen et al., 2016a
Subcortical Stroke	AD-MSC-derived EVs, ~100 nm	100 μg total protein of EVs, intravenously	Rescued cognitive impairments, improved functional recovery and increased axonal sprouting	Otero-Ortega et al., 2017
**NEUROTRAUMA**				
TBI (controlled cortical impact, CCI) in rat	BM-MSC-derived EVs, ~100–150 nm	100 μg total protein of EVs in 500 μl PBS/rat, intravenously	Significantly improved spatial learning, sensorimotor functional recovery, reduced neuroinflammation, increased the number of newly formed mature neurons in the DG, increased the number of newly formed endothelial cells in the lesion boundary zone and DG	Zhang et al., 2015, 2017
TBI (controlled cortical impact, CCI) in mice	BM-MSC-derived EVs, ~200 nm	30 μg total protein of EVs (15 × 10^9^)/mice, single intravenous injection	Rescued cognitive impairments	Kim et al., 2016
Contused SCI in rat	BM-MSC—derived EVs, ~20–130 nm	100 μg total protein of EVs (1 × 10^10^) in 500 μl PBS/rat, intravenously	Decreased expression of proapoptotic protein (Bax) and pro-inflammatory cytokines (TNF-α and IL-1β), increased expression levels of anti-apoptotic (Bcl-2) and anti-inflammatory (IL-10) proteins	Huang et al., 2017
Contused SCI in rat	BM-MSC—derived EVs, ~30–150 nm	40 μg total protein of EVs (1 × 10^6^) in 200 μL PBS/rat, 30 min and 1 day post-SCI, intravenously	Reduced the proportion of A1 astrocytes, decreased lesion area and expression of pro-inflammatory cytokines (TNFα, IL-1α, IL-1β), improved functional recovery	Wang et al., 2018
Contused SCI in rat	BM-MSC—derived EVs	1 × 10^9^ of EVs in 1 ml PBS/rat, 3 h post-SCI, intravenously	Reduced neuroinflammation, decreased reactive microglia and astrocytes, improved functional recovery	Ruppert et al., 2018
Contused SCI in rat	BM-MSC- derived EVs, ~30–100 nm	2.5 × 10^9^ in 200 μl PBS/rat, intravenously	Targeted the SCI site and might contribute to the therapeutic effects, target specifically to M2 macrophages	Lankford et al., 2018

### Multiple Sclerosis (MS)

The success of remyelinating therapy in MS is mostly attributed to dendritic cell (DCs)-derived EVs (Pusic et al., 2014a), whereby EVs produced by IFN-γ-stimulated DCs increased myelination and oxidative tolerance *in vitro* and *in vivo* (Pusic et al., 2014c). These changes are likely attributable to the high levels of miR-219 within the EVs, which is necessary and sufficient for oligodendrocyte precursor cell (OPC) differentiation, the formation and maintenance of compact myelin, and is deficient in human multiple sclerosis lesions (Junker et al., 2009; Dugas et al., 2010; Zhao et al., 2010). Pusic et al. (2014a) proposed that stimulated DCs-derived EVs contain high levels of specific anti-inflammatory miRNAs that may have an immunomodulatory role in suppressing the development of multiple sclerosis. Yu et al. (2013) demonstrated that EVs with membrane-associated TGF-β1 from gene-modified DCs prevented the de novo differentiation of Th17 cells by inhibiting DC IL-6 production, and were effective in inhibiting the development and progression of experimental autoimmune encephalomyelitis (EAE).

Zhuang et al. (2011) used EVs loaded with anti-inflammatory agents like curcumin or JSI124 for intranasal delivery to the brain. Effects of this route of delivery were evaluated in 3 different inflammation-mediated murine disease models: brain tumor-bearing, lipopolysaccharide (LPS)-induced brain inflammation, and EAE. Having given EVs with curcumin, mice were protected from LPS-induced brain inflammation and progression of EAE, brain tumor-bearing mice also showed a significant delay in brain tumor growth following curcumin loaded EV treatment. Intranasal administration of EVs has been shown to provide rapid transport to the brain, and subsequent absorption by the microglia.

A marked reduction in MS and EAE relapses have been observed during pregnancy (Confavreux et al., 1998). It was demonstrated in a mouse model of EAE that serum EVs suppressed T cell activation, promoted the maturation of OPC, and pregnancy-associated exosomes facilitated OPC migration into active CNS lesions (Williams et al., 2013). At the same time, serum EVs derived from non-pregnant mice were also able to reduce the severity of EAE. Thus, it was established that serum EVs are a prominent mediator both of immune modulation and neuroprotection which is especially pronounced during pregnancy.

### Alzheimer's Disease (AD)

Wang et al. (2017) demonstrated the positive effect of using bone marrow MSC-derived EVs (BM-MSC-derived EVs) both *in vitro* and *in vivo*. Inducible nitric oxide synthase (iNOS) mRNA and protein levels were significantly reduced in cultured primary neurons prepared from APP/PS1 pups treated with BM-MSC-derived EVs. *In vivo* studies demonstrated improved cognitive behavior, rescued CA1 synaptic transmission, and long-term potentiation in APP/PS1 mice administrated with BM-MSC-derived EVs through an intracerebroventricular (i.c.v.) injection.

It has already been reported, that neutral endopeptidase (NEP) levels and activity are reduced in patients with AD (Yasojima et al., 2001). NEP was initially identified as a regulator of the Aβ level (Iwata et al., 2000). Katsuda et al. (2013) demonstrated NEP-specific enzyme activity could be exhibited by adipose tissue-derived MSC EVs (AD-MSC-derived EVs). Furthermore, it was indicated that AD-MSC-derived EVs when transferred to N2a cells (a fast-growing mouse neuroblastoma cell line overproducing human Aβ) contributed, at least in part, to a decrease in both extracellular and intracellular Aβ levels. That AD-MSCs expressed NEP at a higher level than BM-MSCs is an important observation highlighting the importance of MSC origin on function (Yasojima et al., 2001).

An alternative cellular source of EVs for therapeutic intervention in AD were used by Yuyama et al. (2015). These authors showed that intracerebral infusion of neuronal EVs into brains of APP transgenic mice decreased amyloid depositions, suggesting an important role for neuronal exosomes in clearing Aβ in brain, but not explaining mechanisms of EVs actions.

### Parkinson's Disease (PD)

Haney et al. (2015) proposed a new EV-based technology for catalase delivery to the CNS to treat PD. Bovine liver derived catalase was loaded into EVs isolated from the mouse macrophage cell line (Raw 264.7). The *in vitro* study data demonstrated that reactive oxygen species (ROS) levels decreased when catalase-loaded EVs as well as empty ones were co-cultured with cortical neurons and dopaminergic (DA) neurons, although the effect of empty EVs was less significant. Administration of the same catalase loaded EVs to a model of 6-OHDA lesions in C57BL/6 female mice had an anti-inflammatory effect manifested as decreased microgliosis. It is important to note that there was no effect on microglia activation, or the number of DA neurons. It was hypothesized that EVs-mediated delivery of catalase to activated microglia, astrocytes, and neurons in the inflamed brain might result in ROS degradation and neuroprotection in PD patients.

Jarmalavičiute et al. (2015) demonstrated *in vitro* that EVs isolated from the supernatants of stem cells derived from the dental pulp of human exfoliated deciduous teeth (SHEDs), and grown on laminin-coated 3D alginate micro-carriers, suppressed 6-OHDA (6-hydroxy-dopamine) induced apoptosis in DA neurons. Interestingly, EVs from the same stem cells cultured under standard conditions possessed no such properties. These results confirm that EVs serve as a novel therapeutic approach in the treatment of Parkinson's disease, although the mechanism of action remains unclear.

### Amyotrophic Lateral Sclerosis (ALS)

Bonafede et al. (2016) showed *in vitro*, that AD-stromal cell-derived EVs (AD-SC-derived EVs) were able to protect NSC-34 cells from oxidative damage, which is one of the main mechanisms of damage in ALS, increasing cell viability. They suggest the positive effect might be caused by secretion of miRNAs, which play a protective role via inhibition of apoptosis pathways, and promoting cell cycle progression and proliferation. AD-SC-derived EVs were shown *in vitro* to ameliorate SOD1 protein levels and aggregates in neuronal cells derived from G93A ALS mice. It was concluded that the abnormal expression of mitochondrial proteins in ALS cells could be rescued by application of AD-SC-derived EVs (Lee et al., 2016).

### Stroke

When using BM-MSC-derived EVs in a model of middle cerebral artery occlusion (MCAO) ischemic lesion volumes reduced, however there was no significant difference compared to PBS-treated controls (Xin et al., 2013a). This study demonstrated that neurite remodeling improved, alongside increases in synaptic plasticity, neurogenesis and angiogenesis within the ischemic boundary zone. These improvements in function were proposed to be the result of miR133b activity on neurite remodeling, delivered to neural cells as part of the BM-MSC-derived EVs cargo (Xin et al., 2013b). In complementary study, intravenous injection of BM-MSC-derived EVs into an *in vivo* model of MCAO reduced post-ischemic motor coordination impairment, induced long-term neuroprotection, increased cell proliferation, and stimulated neurogenesis and angiogenesis (Doeppner et al., 2015).

Chen et al. (2016a) studied the effects of intravenous administration of AD-MSC-derived EVs and AD-MSCs in a model of MCAO. Results demonstrated that neurological impairment was reversed after treatment with AD-MSC-derived EVs an effect that was more significant when treatment used AD-MSCs. Reduced infiltration of CD11+ and CD68+ cells (two indicators of inflammation), decreased oxidative stress and increased angiogenesis were reported in both experimental groups.

Otero-Ortega et al. (2017) demonstrated in a model of subcortical stroke that after intravenous infusion, EVs were found in the brain and peripheral organs (the liver, lungs and the spleen). There was improved functional outcome, increased axonal sprouting, increased oligodendrocyte-associated marker expression and myelin formation, changes in white matter thickness and the restoration of tract connectivity at 28 days after EV administration. Proteomics analysis of the EVs identified 2,416 proteins that are implicated in repairing brain functions.

### Neurotrauma

Zhang et al. (2015) demonstrated that BM-MSC-derived EVs effectively improved functional outcome by promoting endogenous angiogenesis, neurogenesis and reducing inflammation in rats after traumatic brain injury (TBI). In 2 years the same team of scientists reported the results of systemic administration of BM-MSC-derived EVs cultured under 2D and 3D conditions. This treatment did not alter cortical lesion volume but significantly improved cognitive and sensorimotor functional recovery, increased the number of newly formed mature neurons in the dentate gyrus (DG). It also increased the number of newly formed endothelial cells in the lesion boundary zone and DG, as well as reduced neuroinflammation. BM-MSC-derived EVs cultured under 3D conditions provided better outcome in spatial learning compared to EVs from a 2D culture (Zhang et al., 2017).

Kim et al. (2016) demonstrated that BM-MSC-derived EVs suppressed neuroinflammation after TBI in mice. It was also shown that an intravenous infusion of the isolated EVs shortly after induction of TBI rescued pattern separation and spatial learning impairments 1 month later.

Studies of the therapeutic potential of EVs in spinal cord injury (SCI) report that systemic administration of BM-MSC-derived EVs in contused SCI in rats attenuated cell apoptosis and inflammatory processes, stimulated angiogenesis, and promoted functional recovery. In particular, there was a downregulated expression of a pro-apoptotic protein (Bax) and pro-inflammatory cytokines (TNF-α and IL−1β), increased expression levels of anti-apoptotic (Bcl-2) and anti-inflammatory (IL-10) proteins (Huang et al., 2017). Furthermore, Lankford et al. (2018) demonstrated that intravenously delivered BM-MSC-derived EVs targeted the SCI site, might contribute to the therapeutic effects by specifically targeting M2 macrophages. EVs were detected in the area of SCI and the spleen, but not in the intact region of the spinal cord.

Recently, Wang et al. (2018) confirmed a comparable therapeutic effect of intravenously administered BM-MSC-derived EVs and BM-MSCs in SCI. The authors observed that BM-MSC-derived EVs reduced the proportion of neurotoxic astrocytes, probably via inhibiting nuclear translocation of NFκB p65, and exerted anti-inflammatory and neuroprotective effects following SCI. Similar results were obtained by Ruppert et al. (2018), who confirmed the anti-inflammatory and functional effects of intravenously delivered BM-MSC-derived EVs by means of a decreased in microglia activation markers rather than skewing microglial polarization.

### Clinical Trials Using EVS in the Treatment of Nerve Disorders

The conceptual advance in our understanding of the therapeutic application of EVs as a regenerative strategy for neural tissue is relatively new. Therefore, a considerable number of studies are non-clinical, relying on *in vitro* and *in vivo* models. To date the Food and Drug Administration (FDA) have approved clinical trials using EVs as a treatment of ulcers (ClinicalTrials.gov Identifier: NCT02565264), sepsis (NCT02957279), macular hole (NCT03437759), and type 1 diabetes mellitus (NCT02138331). Based on pre-clinical studies of EV-mediated delivery of miR-124 promoting neurogenesis after ischemia (Yang et al., 2017), a further clinical trial using MSC-derived EVs in the treatment of acute ischemic stroke (NCT03384433) is planned for 2018–19, in which the effect of MSC-generated EVs will be examined in post-stroke phases 1 and 2.

## Conclusion

Studies of the therapeutic potential of EVs in regenerative strategies strongly support the use of MSC-derived EVs in the treatment of nerve tissue disorders. MSC-derived EVs are reported to exert the majority of the positive effects seen with the direct use of MSCs as a therapy, but without the risks associated with stem cell transplantation (Gatti et al., 2011; Li et al., 2012; Konala et al., 2016). The rapid progression from non-clinical to clinical studies of MSC-derived EVs will uncover the exciting potential that EV-mediated cell free therapy may offer for the treatment of nerve disorders.

## Author Contributions

LG: collection of data on the characteristics of immunomodulatory and neuroprotective effects of EVs, compilation these data. VJ: collection of data on the transplantation EVs in neurodegeneration deseases, professional English editing. YM: collection of data on the transplantation EVs in stroke and traumatic injury, compilation these data, drawing Figure 1. AR: compilation of article content, writing some chapters.

### Conflict of Interest Statement

The authors declare that the research was conducted in the absence of any commercial or financial relationships that could be construed as a potential conflict of interest.
